# Global Diversity of the Placozoa

**DOI:** 10.1371/journal.pone.0057131

**Published:** 2013-04-02

**Authors:** Michael Eitel, Hans-Jürgen Osigus, Rob DeSalle, Bernd Schierwater

**Affiliations:** 1 Stiftung Tierärztliche Hochschule Hannover, Institut für Tierökologie und Zellbiologie, Ecology and Evolution, Hannover, Germany; 2 The Swire Institute of Marine Science, Faculty of Science, School of Biological Sciences, The University of Hong Kong, Hong Kong; 3 Sackler Institute for Comparative Genomics and Division of Invertebrate Zoology, American Museum of Natural History, New York, New York, United States of America; 4 Department of Molecular, Cellular and Developmental Biology, Yale University, New Haven, Connecticut, United States of America; Institute of Marine Research, Norway

## Abstract

The enigmatic animal phylum Placozoa holds a key position in the metazoan Tree of Life. A simple bauplan makes it appear to be the most basal metazoan known and genetic evidence also points to a position close to the last common metazoan ancestor. *Trichoplax adhaerens* is the only formally described species in the phylum to date, making the Placozoa the only monotypic phylum in the animal kingdom. However, recent molecular genetic as well as morphological studies have identified a high level of diversity, and hence a potential high level of taxonomic diversity, within this phylum. Different taxa, possibly at different taxonomic levels, are awaiting description. In this review we firstly summarize knowledge on the morphology, phylogenetic position and ecology of the Placozoa. Secondly, we give an overview of placozoan morphological and genetic diversity and finally present an updated distribution of placozoan populations. We conclude that there is great potential and need to erect new taxa and to establish a firm system for this taxonomic *tabula rasa*.

## Introduction

This review was prompted by the World Register of Marine Species ([1]; see PLOS ONE WoRMS collection at http://www.ploscollections.org), of which the World Placozoa Database (http://www.marinespecies.org/placozoa/) is a part.

### *Trichoplax adhaerens* and the phylum Placozoa

**Figure 1:**
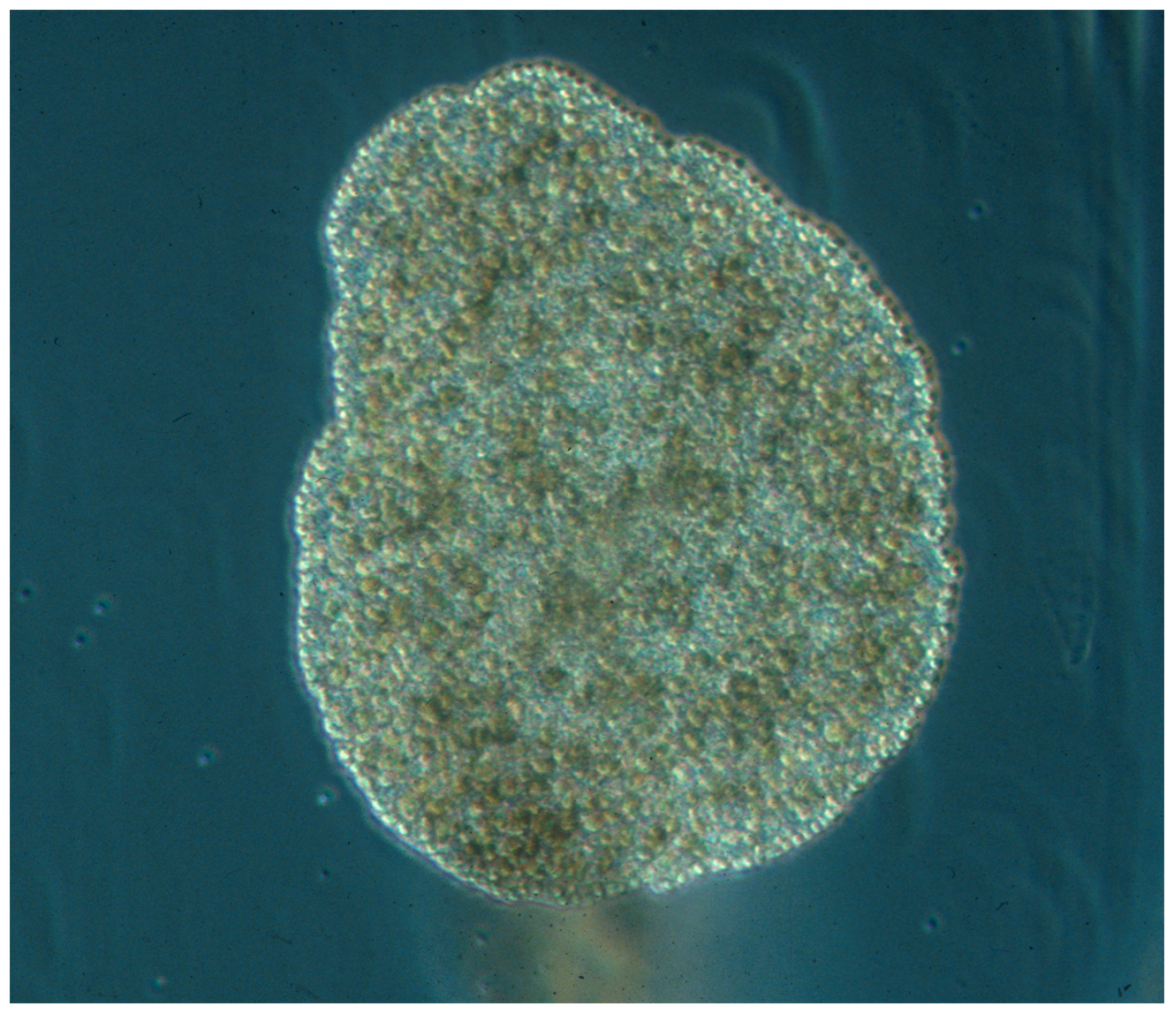
Photograph of *Trichoplax adhaerens*, F.E. Schulze (1883). The animal is about 0.5 mm in diameter. Photo by B. Schierwater, for additional images and videos of placozoan specimens see www.trichoplax.com and www.placozoa.de.

In the late 19th century Franz Eilhard Schulze, a German zoologist, identified a small (1–3 mm) amoeba-like animal crawling on the sides of an aquarium at Graz University containing marine samples from the bay of Trieste (Italy). He soon realized that this animal did not fit any of the known metazoan bauplans, and studied the small creatures in detail using various microscopy techniques. According to its flat appearance, the ciliated surface and the observation that the animal stuck to the surface, he named the new species *Trichoplax adhaerens* (Greek “tricha" = ‘hair’ and “plax" = ‘plate’, Latin “adhaerere" = ‘to stick’; see Figure 1), the ‘sticky hairy plate’. He published an initial short description in 1883 [2]. Eight years later, in 1891, Schulze published a more comprehensive description of *Trichoplax adhaerens* [3] with detailed drawings of its structure, and most of Schulze's early observations are still valid today.

Shortly after the discovery of *Trichoplax adhaerens* research on this enigmatic species ceased. Karl Gottlieb Grell ‘rediscovered’ *Trichoplax* after nearly a century and, together with colleagues, studied its ultrastructure and observed sexual reproduction in *Trichoplax* [4]–[32]. According to Grell's studies, *Trichoplax adhaerens* was so different from all other known animal taxa that it deserved its own phylum. Grell named this phylum “Placozoa", from Bütschli's ‘Placula’ - a hypothetical two-layered and benthic ‘Urmetazoon’ ([7], [33]; for a historical summary of placozoan research see [34]–[36]). More than a century after the discovery of *Trichoplax adhaerens* the phylum status of the Placozoa – as envisioned by Schulze – was finally accepted.

It is important to note that animals described as *Trichoplax adhaerens* in Grell and colleagues' studies might, although being morphologically highly similar to Schulze's *Trichoplax*, actually belong to a different species. Given our current knowledge of placozoan diversity (see below) it is evident that the various studies of placozoans accomplished over the years have pooled a broad diversity of taxa under the single species ‘*Trichoplax adhaerens*’.

### The morphology of *Trichoplax adhaerens sensu* Grell & Benwitz

Based on Schulze's early morphological studies and Grell and colleagues' ultrastructural research [2], [3], [5], [13], [17], [19]–[22], [24], [26], [28] *Trichoplax* possesses a three-layered sandwich organization with morphologically different upper (protective) and lower (nutritive) epithelia. These two layers enclose a meshwork of connected fiber cells that are responsible for the changes in shape of *Trichoplax*. The lower epithelium is specialized for extracellular digestion and consists of small gland cells and relatively large columnar cells. Cells of both epithelia are monociliated, those of the lower epithelium mediate ciliary walking. Belt desmosomes are the only linkage between the loosely connected epithelial cells and the typical metazoan basal lamina and extracellular matrix ( = ECM) are lacking at least structurally. It is interesting to note, however, that genes associated with an ECM and cell adhesion have been identified in the genome of *Trichoplax adhaerens* [37]. These observations, together with the fact that *Trichoplax* lacks axes of symmetry (the top-bottom orientation has polarity but not a defined axis) make *Trichoplax* the most simply organized metazoan animal known.

**Figure 2:**
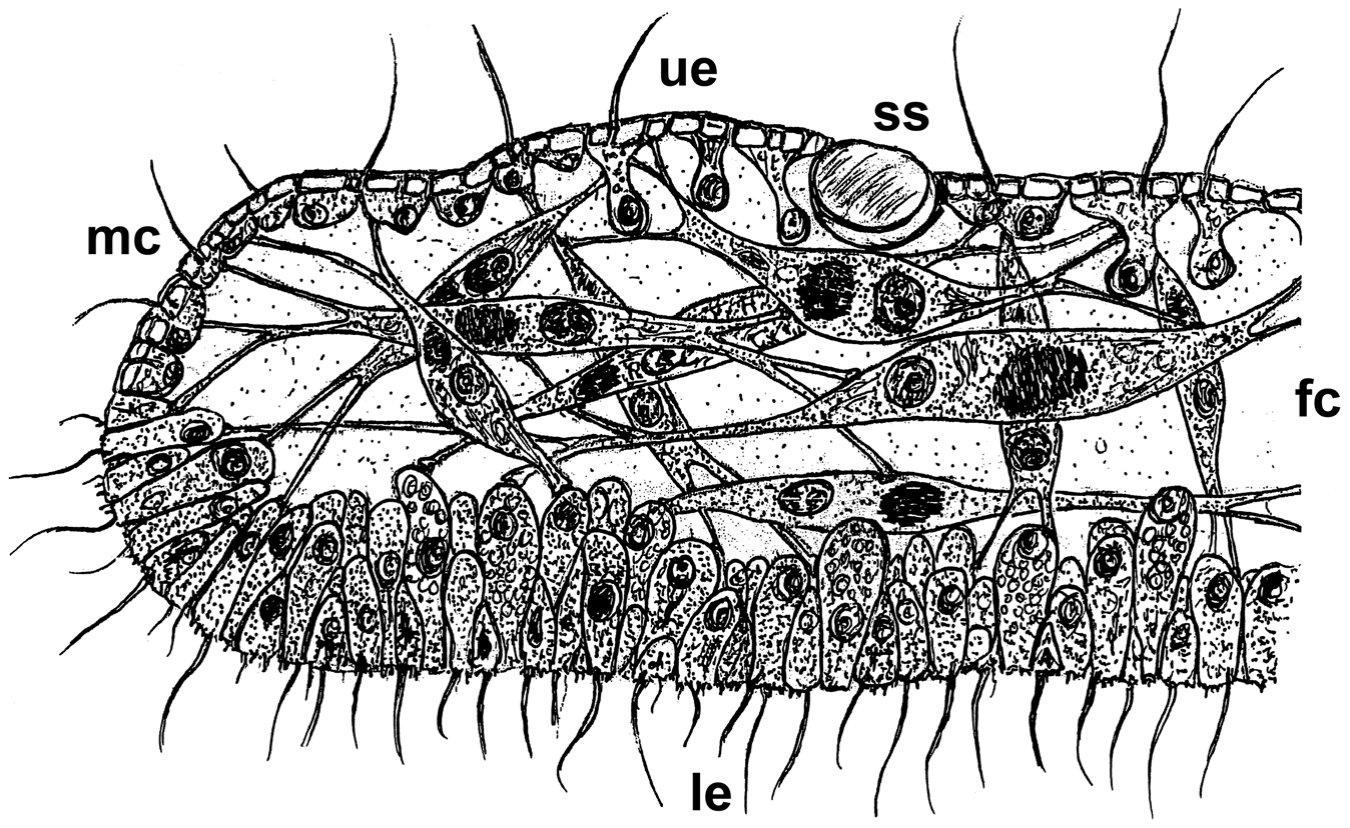
Revised schematic cross sections of a Placozoon. Placozoans possess two epithelia, an upper epithelium facing the water and a lower epithelium facing the substrate. The traditional schematic cross section of a Placozoon [4] shows a single fiber cell layer sandwiched between these layers. Recent studies by Guidi et al. [38], however, have shown that all morphologically studied genetic lineages, including the lineage originally used by Grell and Benwitz [17], actually possess several fiber cell layers. The given drawing thus represents the revised cross section of a typical placozoan specimen. It was also previously shown that the small marginal cells might represent the formerly described pluripotent stem cells [38]. ue = upper epithelium; le = lower epithelium; fc = fiber cells; mc = marginal cells; ss = shiny sphere.

A recent study by Guidi *et al.*, 2011 [38] revealed first insights into morphological differences among various placozoan lineages and substantially amended our knowledge of traditional morphology. The authors showed that the fiber cells are not arranged in one layer only, but in a 3D meshwork with different subtypes of fiber cells. In addition, a fifth cell type was identified in this study. This cell type was previously identified based on functional genetic studies by Jakob *et al.*, 2004 [39], where the authors found an expression of the Hox/ParaHox gene *Trox-2* at the margin of the animal. Using ultrastructural studies Guidi *et al.* [38] identified a unique cell type in the position of previously postulated stem cells. Based on these new results a revised bauplan was proposed for the Placozoa (Figure 2).

### Placozoa - relationships to other animal phyla

All animals on the planet, however diverse, are descended from a last common metazoan ancestor (LCMA). Due to a lack of fossil evidence, we can only speculate about what the first metazoan was like, and how evolution proceeded from this LCMA to extant metazoan taxa. Understanding the phylogenetic position and taxonomic status of the phylum Placozoa is, therefore, fundamental to addressing questions about the origin of the Metazoa.

**Figure 3:**
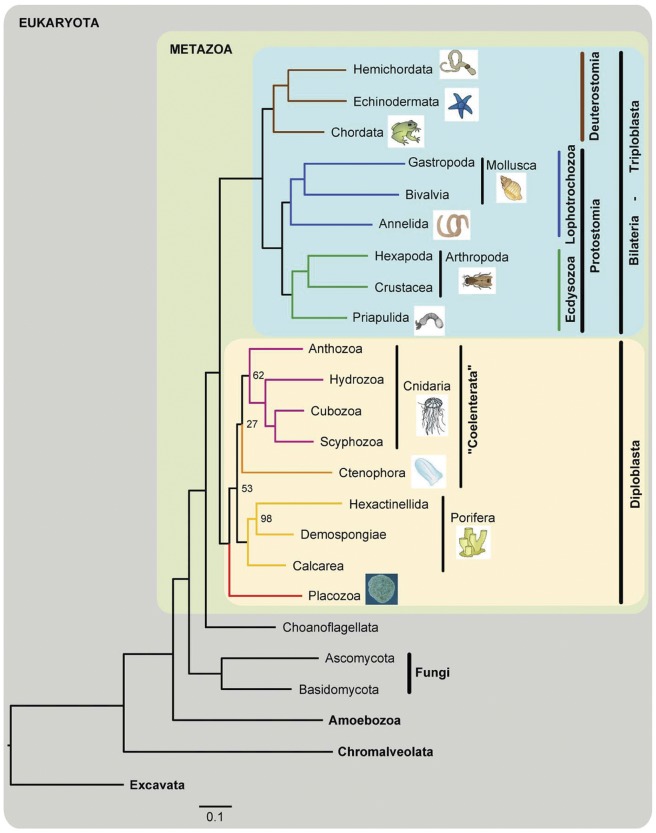
Maximum likelihood phylogenetic tree of metazoan relationships using a concatenated data matrix. In this tree Placozoa group basal within the diploblasts (like in many other studies) but diploblasts and triploblasts occur as sister groups, a hypothesis that fits many morphological and structural genetics characters but is highly controversially discussed. From Schierwater *et al.*, 2009 [44].

Historically, placozoans have been placed at the very base of the metazoan Tree of Life because of their very simple morphology. Early molecular phylogenetic studies based on nuclear small and large rRNA subunits (18S and 28S) have, however, placed *Trichoplax* at various positions, including as a sister taxon to the (i) Cnidaria, (ii) Ctenophora, and (iii) even to Bilateria (reviewed in [40]). A recent study using 106 genes from the complete nuclear genome placed *Trichoplax* basal to Eumetazoa, with Porifera branching off first [37]. Recently, a similar phylogeny has been reported based on the first sponge genome [41]. In contrast to this commonly proposed ‘Porifera-basal-model’, mitochondrial genome and total evidence based analyses found an early evolutionary split into two sister clades: the ‘Bilateria’ (or triploblasts) and the ‘non-Bilateria’ (or diploblasts) (Figure 3; [42]–[45]). The latter comprises the animal phyla Placozoa, Porifera, Cnidaria and Ctenophora, with placozoans being basal within this clade [43]–[45]. More recent studies show very low, or even no, support for any of the above hypotheses and illustrate the problems of molecular systematic analyses of large data sets in the basal metazoans [46], [47]. Molecular data alone, with thousands of genes or even whole genomes, might not be sufficient to address the question of basal metazoan relationships. This issue will most likely be resolved from a combination of all available data from various fields including morphology, development, molecular morphology (secondary structure of various molecules), mitochondrial genome data, and also from whole genome comparisons (see e.g. [48]).

### Collecting placozoans

To sample placozoans two different methods are traditionally used, namely ‘rock sampling’ and ‘slide sampling’ [49]–[51]. The rock sampling method employs the collection of stones and other hard substrates, such as dead parts of scleractinian corals and mussel shells at depths up to 5 m. The slide sampling technique uses standard microscopic glass slides (76×26 mm), which are housed in plastic slide containers, cut open at the top and the bottom to enable the flow-through of seawater and allow establishment of a biofilm on the slides, a settling ground for placozoans. The racks are connected with nylon ropes and attached in suitable placozoan habitats at depths between 2–20 m. After a few days to three weeks, depending on the season and the sampled habitat (see [49], [50] for details) the slides are collected and placozoans isolated from the slides.

Besides these standard ‘natural environment’ sampling methods another highly valuable source for collecting placozoans are seawater aquaria. Geographic assignment of placozoans found in aquaria is in most cases impossible as stones and corals in aquaria are commonly a mixture of different samples from various places. Aquaria are nonetheless helpful for screening genetic diversity within the Placozoa. Several publications have, for example, reported finding placozoans in cultures intended to study other marine organisms [52]–[54]. Given the large number of scientific marine stations, the screening of their aquaria inventory would surely lead to the identification of a large number of placozoan specimens and potential new species.

### Ecology

Placozoans have been found in coastal waters up to a depth of 20 m [49], [50]. Preferred habitats seem to be calm water areas with hard substrata (stony beaches, rocky shores, coral reefs, mangrove roots), whereas they are less abundant in areas with sandy surfaces and strong wave activity [13], [49], [50], [55]. Specimens have been found in the tropics, with water temperatures of up to 32°C but also at locations with much lower temperatures (just above 10°C) in the winter (e.g., the Mediterranean Sea) [56]. Placozoans inhabit highly diverse ecological systems, such as intertidal rocky pools, muddy water ponds, coral reefs, mangroves, flow through seawater systems, boat docks/harbors and stony beaches (see [49], [50] and references therein). These different habitats provide highly diverse environmental conditions, which may contribute to the ecological speciation in the phylum. These habitats can show large temporal and spatial variation in conditions; harbors for example are mostly calm areas with reduced turbidity, but are directly influenced by anthropogenic factors. Mangrove streams often contain brackish water, and placozoans in these habitats have to deal with rapid changes in salinity, which is also an important factor in areas receiving heavy rainfall. Living animals collected in Hong Kong in September 2007, for example, were isolated from seawater with a salinity of only 20 ppt, and laboratory observations have shown that placozoans can tolerate continuously increasing salinity up to 50 ppt and, in rare cases, even to 60 ppt (Eitel *et al.*, unpublished).

Placozoans also show seasonality in their life history patterns. Generally placozoans are more abundant in warmer months, i.e. between June and October in subtropical and temperate regions in the Northern Hemisphere. In Shirahama (Japan) placozoan abundance peaked at late summer and individuals were found on more than 80% of all sampling slides and on over 60% of ‘rock samples’ [57]. Adult placozoans were found year-round, even in the wintertime. In Hong Kong a low number of placozoans were recorded during winter and before summer (December–April), whereas in the summer months (May–October) many individuals were collected with >90% positive slide racks and occasionally more than 200 individuals on a single slide rack ([49]; Eitel *et al.*, unpublished).

Preliminary observations on the biotic interactions of placozoans suggest co-occurrence with filter feeders, such as sessile ciliates and polychaetes [50]. Because of their small size and benthic lifestyle placozoans should be prone to predation, but so far only a single instance of predation has been recorded [58]. Cases of potential predators (rhabdocoels, serpulids, gastropods and cnidarians) being repelled after contact with placozoans suggest the possibility of a chemical defense mechanism, probably mediated through lipid-filled droplets (‘shiny spheres’) of the upper epithelium [36], [50], [59].

### Diversity in a monotypic phylum?

Until a few years ago it was presumed that *Trichoplax adhaerens* was the only species within the phylum Placozoa, since all placozoans found worldwide looked very similar under light microscopy. Voigt *et al.* [51], however, showed that the monotypic phylum Placozoa harbors substantial genetic variation among well-isolated lineages. Obviously there is a large amount of morphologically cryptic forms and, as we discuss below, genetic diversity in placozoans. We will next address existing morphological and genetic differences among placozoans.

## Results and Discussion

### Morphological diversity

Morphological studies are an important initial step to identifying new species in the phylum Placozoa. In one of the first studies addressing morphological differences among different placozoan isolates, Guidi *et al.* [38] used scanning and transmission electron microscopy to compare ten different clonal lineages. Eight morphological characters were identified that allowed the grouping of these clonal lineages into five morpho-groups (Table 1). Ultrastructural differences were identified in the upper and lower epithelium as well as the fiber cell layer. Exemplary, we here focus on morphological features that separate two morpho-groups from the remaining placozoans. The so-called ‘GRELL’ lineage differs from the other four morpho-groups by the existence of unique microtubules in the cells of the upper epithelium. The ‘MEDI’ lineage shows a unique structure of the mitochondrial complex – a structure of unknown function within the fiber cells that is built up of alternating layers of mitochondria and vesicles [17]. Only the ‘MEDI’ lineage shows a very high density of the mitochondrial matrix and the vesicles are thin and electron-dense. All other lineages show a matrix with a low density and vesicles are large and electron-transparent. Further studies are, however, needed to determine at which taxonomic level the given characters are diagnostic.

**Table 1:** Diagnostic morphological characters identified in the Placozoa.

	CLONE NAME	GRELL	TUN-A	HWH-A	HWH-B	PAN	TUN-B	OKH-A	KEN-A	TEN-A	MEDI
	CLONAL LINEAGE GROUP	I	II	II	II	III	III	III	III	IV	V
	**UPPER EPITHELIUM**										
A1	Microtubules	1	0	0	0	0	0	0	0	0	0
	0: Absent										
	1: Present										
A2	Cellular surface	0	0	0	0	1	1	1	1	1	1
	0: Poligonal										
	1: Rounded										
A3	Cell arrangement	0	0	0	0	1	1	1	1	1	1
	0: Juxtaposed cells										
	1: Separated cells										
A4	Concave disc	0	0	0	0	1	1	1	1	1	1
	0: Absent										
	1: Present										
A5	Desmosomes	1	1	1	1	0	0	0	0	0	1
	0: Low number										
	1: High number										
	**LOWER EPITHELIUM**										
B	Secreted material	0	0	0	0	0	0	0	0	1	0
	0: Not evident										
	1: Abundant										
	**FIBER CELLS**										
C1	Mitochondrial matrix	1	0	0	0	0	0	0	0	0	1
	0: Low density										
	1: High density										
C2	Mitochondrial complex vesicles	0	0	0	0	0	0	0	0	0	1
	0: Large and electron-transparent										
	1: Thin and electron-dense										

Eight distinctive ultrastructural characters from the upper epithelium (A1–A5), the lower epithelium (B), and the fiber cells (C1–C2) allow distinguishing five morphological groups (I–V). Modified from Guidi *et al.*, 2011 [38].

Mapping of morpho-groups to ecological parameters has not resulted in a conclusive picture that enables us to establish new taxonomic units using this diagnostic approach [60]. Additional characters from other disciplines, such as molecular genetics, are required to complete this picture.

### Genetic diversity – Single marker gene analyses

Over the last decade, substantial efforts have been made to unravel the molecular diversity within the Placozoa. The first study to characterize molecular differences between different clonal lineages was published in 2004 by Aleoshin *et al.* [61]. The authors identified very little variation between the so-called ‘GRELL’ lineage of *Trichoplax adhaerens* and a *Trichoplax* sp. lineage from the Russian coast of the Japanese Sea. Some slight morphological differences were observed between the two lineages, which also showed some sequence differences in the large mitochondrial and nuclear ribosomal subunits (16S and 28S). Fourteen positions were unequivocally substituted in the 16S fragments (2% divergence in 703 bp) between *Trichoplax adhaerens* and *Trichoplax* sp. In the 28S only two substitutions were found in a 299 bp fragment (<0.7% sequence divergence). Aleoshin *et al.* [61] concluded that the genetic distance between the two lineages was too low to warrant separation into different species and that both lineages should be considered as *Trichoplax adhaerens*. Our current perspective (see below), however, offers a different interpretation, especially in the light of unresolved base pairs in the 16S sequence published by Aleoshin *et al.* [61].

In the first comprehensive study of placozoan genetic diversity, Voigt *et al.*, 2004 [51] used four molecular markers to identify the diversity within the Placozoa. In this study 31 different isolates were compared for the 16S, 18S, 28S and ITS loci. Five clearly separated clades were identified, harboring eight distinct haplotypes. For the nuclear 18S ribosomal DNA marker, the observed genetic distances between the most distantly related placozoan haplotypes are comparable to distances between families within orders in other basal metazoan phyla (e.g., the Porifera, Cnidaria and Ctenophora) and clearly indicates divergence events in the Placozoa far beyond the genus level. The authors also provided data on the secondary structure of 16S rRNA, which further support a high diversity in the Placozoa.

A third study addressing the diversity of the Placozoa based on samples from the coasts of five Caribbean nations was published by Signorovitch *et al.*, 2006 [55]. Seven out of eight formerly described 16S haplotypes plus two new haplotypes were identified (named H4-2 and H4-3 therein and renamed to H9 and H10 in [50]). All but one of the known clades were found (just clade IV, H5 was missing).

Pearse & Voigt, 2007 [50] added new partial 16S sequences from two different locations: the Monterey Bay aquarium (California, USA) and Lizard Island (Australia). The Monterey Bay aquarium samples yielded two new haplotypes, H11 and H17 [50], [62]. The H11 haplotype was highly divergent from the existing clades and the new clade VI was therefore established [49]. The Lizard Island samples all belonged to the 16S haplotype H9 (clade V).

Eitel & Schierwater published what is currently the largest survey of placozoan specimens in 2010 [49]. A total of 39 tropical and subtropical locations were screened for placozoans, of which 23 yielded placozoans that were isolated and genotyped, almost doubling the overall number of genetically characterized placozoan populations (locations). Seven of the eleven haplotypes that were known at that time were found in the samples. In addition, five new 16S haplotypes were found, named Placozoa spp. H12-H16. Four of these (H13-H16) belong to the formerly named clades III and V. The extreme genetic divergence of the H12 lineage, however, required the establishment of a new clade, clade VII.

**Figure 4:**
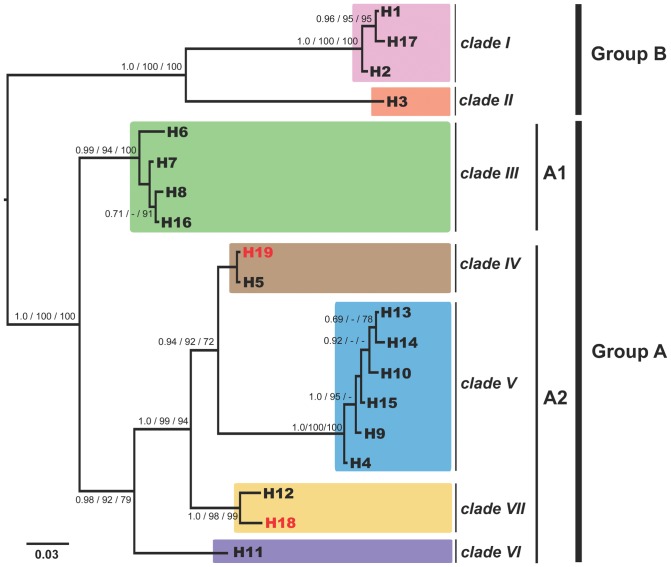
Unexpected diversity has been found in the phylum Placozoa. Shown is the 16S Bayesian inference phylogram of different placozoan haplotypes. Presently seven genetically highly different clades (I–VII) have been identified. Haplotypes H18 and H19 have been newly identified in this study (red). Please note that the lineages H5 and H19 differ in just one nucleotide position within a highly variable loop region, which has been removed from the alignment (the result is the shown polytomy). Current knowledge on placozoan biodiversity is still limited and more samples are urgently needed. For details on phylogenetic analyses see Material and Methods and [49]. Numbers beside nodes are from left to right: Bayesian posterior probabilities, Maximum likelihood and Maximum Parsimony bootstrap support. Values below 70% are marked with ‘-’.

**Figure 5:**
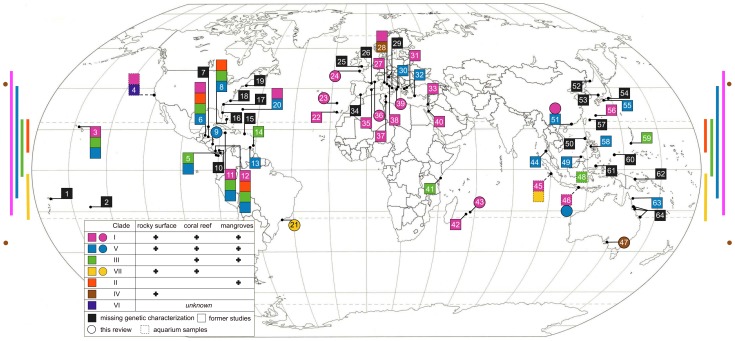
Worldwide distribution of genetically characterized placozoan specimens. The ten newly genotyped sites from this study are marked as circles and known genotypes from other studies are marked as squares. Samples from aquaria or holding tanks (‘aquarium samples’) with only a presumed/guessed origin are labeled with dashed lines. Note that several numbers combine multiple sampling sites. The color-coded bars and spots on the left and right mark the latitudinal distribution of the clades with at least two isolates from different locations each. Clades with a higher latitudinal distribution are also found in more habitats (see legend inlet). Modified from Eitel & Schierwater, 2010 [49]. 1. Western Samoa; 2. Moorea, French Polynesia; 3. Oahu, Hawaii, US; 4. Monterey Bay, California, US (A.s.); 5. Pacific coast of Panama; 6. Twin Cays, Belize; 7. Puerto Morelos, Mexico and Roatan, Honduras; 8. Bahamas; 9. Cahuita, Costa Rica; 10. Galeta, Panama; 11. Discovery Bay, Jamaica; 12. Bocas del Toro, Panama; 13. Cubagua, Venezuela; 14. Grenada; 15. Puerto Rico; 16. Florida, US; 17. North Carolina, US; 18. Philadelphia, US; 19. Woods Hole, US; 20. Bermuda, GB; 21. Sao Sebastiao Channel, Brazil; 22. Puerto de la Cruz, Tenerife, Spain; 23. Quinta do Lorde Marina, Madeira, Portugal; 24. Roscoff, France; 25. Plymouth, GB (A.s.); 26. Banyuls-sur-Mer, France; 27. Castiglioncello, Italy; 28. Orbetello Lagoon, Italy; 29. Gulf of Trieste and Gulf of Naples, Italy; 30. Otranto, Italy; 31. Katerini and Ormos Panagias, Greece; 32. Turunc, Turkey; 33. Caesarea, Israel; 34. Almunecar, Granada, Spain; 35. Cala Rajada, Majorca, Spain ; 36. Cassis, France; 37. Yasmine and Zarzis, Tunisia; 38. San Felice Circeo, Italy; 39. Porto Cesareo, Italy; 40. Elat, Israel; 41. Mombasa, Kenya; 42. Reunion ; 43. Mauritius, France; 44. Laem Pakarang, Thailand; 45. Indonesia (A.s.); 46. Bali, Indonesia; 47. Adelaide, Australia; 48. ‘Indo-Pacific’ (A.s.); 49. Kota Kinabalu, Sabah, Malaysia ; 50. Zambales, Philippines; 51. Hong Kong, China; 52. Russian coast of the Sea of Japan; 53. Oki Island, Japan; 54. Shimoda, Honshu, Japan; 55. Shirahama, Honshu, Japan; 56. Chatan, Okinawa, Japan; 57. Iriomote, Ryukyu Islands, Japan; 58. Boracay, Philippines; 59. Guam, US; 60. Palau; 61. Manado, Sulawesi, Indonesia; 62. Madang, Papua New Guinea; 63. Lizard Island, Australia; 64. Townsville, Orpheus Island and Heron Island, Australia.

**Figure 6:**
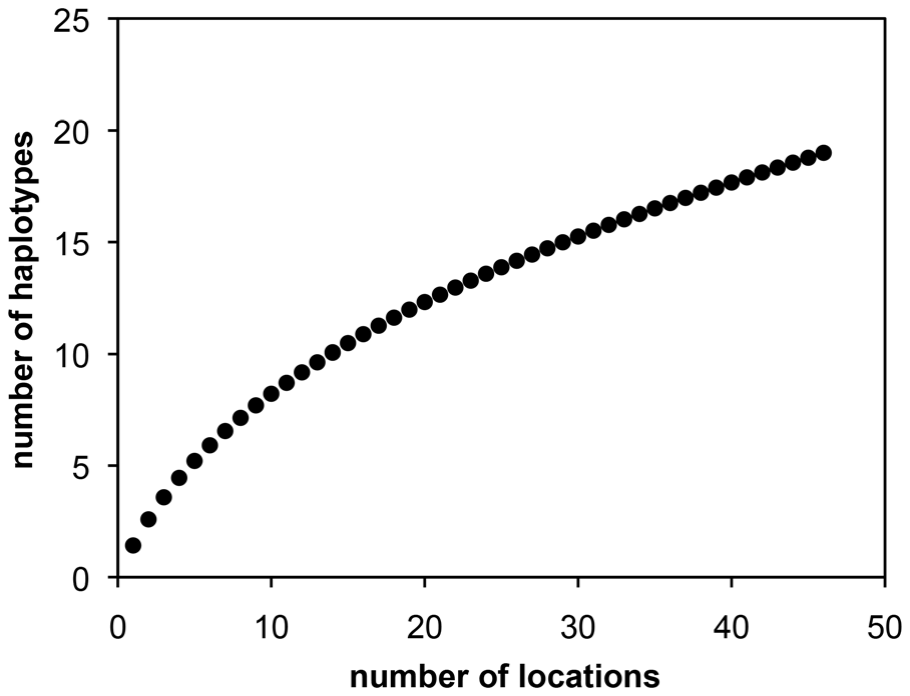
Coleman rarefaction curve. The total number of different haplotypes is plotted against the number of genetically screened locations. A slight saturation effect is apparent starting at seven locations and eight haplotypes. The slope of the curve is, however, artificially reduced as continuously re-sampling of the two most frequent lineages occurs (H2 and H4). Both haplotypes together have been found in 47% of all genetically characterized locations. Extrapolation of the curve estimates the total number of placozoan haplotypes (species) to be higher than some 200.

Since this initial phylogeographic study [49] subsequent sampling has revealed additional placozoan isolates from ten new localities (see Table 2 and Figures 4 and 5). 16S genotyping revealed the presence of already known haplotypes in eight of the isolates: six H2 samples (clade I) and two H4 samples (clade V). Samples from Brazil yielded the new haplotype H18, a sister taxon of the H12 lineage in clade VI, increasing the number of haplotypes in this clade to two (Figure 4). The four specimens isolated from southern Australia belong to haplotype H19, a new taxon in clade IV. To estimate haplotype completeness, a Coleman rarefaction curve was calculated using all samples from Table 2 (Figure 6). The new estimate of expected species diversity in placozoans was estimated at ∼200 genetic lineages, which is close to an earlier estimate published in Eitel & Schierwater [49].

**Table 2:** Locations of genotyped placozoans.

Oceanographic Region	Clade	Haplotype	Sampling site	Genotyped isolates	Habitat type	No. in Fig. 5	Date of collection	Reference
C Pacific Ocean	I	H1	Oahu, Hawaii, US	?	?	3	?	[50]
		H2	Oahu, Hawaii, US	?	?	3	?	[50]
	III	H6	Oahu, Hawaii, US	?	?	3	?	[50]
		H8	Oahu, Hawaii, US	1	boat dock	3	05/2007	[49]
	V	H4	Oahu, Hawaii, US	?	?	3	?	[50]
E Pacific Ocean	I	H1	Isla Iguana, Panama, Panama	1	?	5	2002	[51]
		H2	Monterey Bay, California, US (A.s.)	?	A.s.	4	?	[50]
		H17^a^	Monterey Bay, California, US (A.s.)	?	A.s.	4	?	[50]
	III	H6	Isla Iguana, Panama, Panama	2	?	5	2002	[51]
		H7	Naos Island Laboratory, Panama	1	?	5	2002	[51]
		H8	Isla Iguana, Panama, Panama	2	?	5	2002	[51]
	V	H4	Naos Island Lab, Panama	1	?	5	2002	[51]
		H4	Achitones Laboratory, Panama	3	A.s.	5	2003	[51]
	VI	H11	Monterey Bay, California, US (A.s.)	2	A.s.	4	?	[50]
Caribbean	I	H1	Discovery Bay, Jamaica	1	boat dock	11	02/2003	[55]
		H1	Bocas del Toro, Panama	1	?	12	2002/2003	[51]
		H2	Twin Cays, Belize	5	mangroves	6	08/2002	[55]
		H2	Bocas del Toro, Panama	6	mangroves	12	2002/2003	[51], [55]
	II	H3	Twin Cays, Belize	5	mangroves	6	08/2003, 06/2004	[55]
		H3	Bahamas	1	flow-through seawater system	8	2001	[49]
		H3	Bocas del Toro, Panama	1	?	12	2002/2003	[51]
	III	H6	Twin Cays, Belize	3	mangroves	6	08/2003, 06/2004	[55]
		H6	Grenada	1	boat dock	14	03/2003	[55]
		H6	Grenada	1	mangroves	14	03/2003	[55]
		H7	Twin Cays, Belize	8	mangroves	6	08/2003, 06/2004	[55]
		H8	Twin Cays, Belize	9	mangroves	6	08/2002, 08/2003, 06/2004	[55]
		H8	Bocas del Toro, Panama	1	mangroves	12	2002/2003	[55]
		H8	Bahamas	1	flow-through seawater system	8	2001	[49]
		H8	Discovery Bay, Jamaica	2	boat dock	11	02/2003	[55]
	V	H4	Twin Cays, Belize	9	mangroves	6	08/2002, 08/2003, 06/2004	[55]
		H4	Cahuita, Costa Rica	1	stony beach	9	04/2010	this review
		H4	Bocas del Toro, Panama	3	?	12	2002/2003	[51]
		H4	Cubagua, Venezuela	1	?	13	2002	[51]
		?^b^	Discovery Bay, Jamaica	1	?	11	?	[63]
W Atlantic Ocean	I	H2	Bermuda, GB	1	mangroves	20	08/2005	[55]
	V	H4	Bermuda, GB	1	mangroves	20	08/2005	[55]
		H9	Bermuda, GB	4	open pond	20	08/2005	[55]
		H9	Bermuda, GB	2	mangroves	20	08/2005	[55]
		H10	Bermuda, GB	4	open pond	20	08/2005	[55]
		H10	Bermuda, GB	2	mangroves	20	08/2005	[55]
		H10	Bermuda, GB	1	boat dock	20	08/2005	[55]
	VII	H18	Sao Sebastiao Channel, Brazil	1	stony beach	21	10/2009	this review
E Atlantic Ocean	I	H2	Puerto de la Cruz, Tenerife, Spain	6	stone pool	22	08/2007	[49]
		H2	Quinta do Lorde Marina, Madeira, Portugal	3	boat dock	23	05/2011	this review
		H2	Roscoff, France	1	flow-through seawater system	24	05/2009	[79], this review
Mediterranean Sea	I	H1	Cala Rajada, Majorca, Spain	1	stone pool	35	10/2006	[49]
		H2	Katerini, Greece	2	boat dock	31	08/2008	[49]
		H2	Ormos Panagias	1	boat dock	31	05/2009	[49]
		H2	Cassis, France	1	boat dock	36	06/2011	this review
		H2	Yasmine,Tunisia	3	boat dock	37	04/2006	[49]
		H2^c^	Castiglioncello, Italy	4	stony beach	27	05/2008	[49]
		H2	Caesarea, Israel	8	stony beach	33	01/2007	[49]
		H2	Zarzis, Tunisia	4	stony beach	37	07/2008	[49]
		H2^c^	Porto Cesareo, Italy	1	stony beach	39	09/2010	this review
		H2	Orbetello Lagoon, Italy	4	open pond/lagoon	28	10/2003	[51], [56]
		H2^c^	San Felice Circeo, Italy	2	open pond	38	10/2007	[49]
	IV	H5	Orbetello Lagoon, Italy	3	open pond/lagoon	28	10/2003	[51], [56]
	V	H9	Turunc, Turkey	3	stony beach	32	08/2007	[49]
		H10^c^	Otranto, Italy	4	stony beach	30	08/2008	[49]
Red Sea	I	H1	Elat, Israel	1	algae bed	40	1978	[17], [51]
Indian Ocean	I	H2	Reunion	4	coral reef	42	12/2006	[49]
		H2	Mauritius, France	1	stony beach	43	10/2010	this review
	III	H16	Mombasa, Kenya	2	coral reef	41	05/2007	[49]
	IV	H19^c^	Adelaide, Australia	2	stony beach	47	05/2011, 12/2011	this review
	V	H4	Laem Pakarang, Thailand	3	stony beach	44	03/2008	[49]
Indo-Pacific	I	H2	Indonesia (A.s.)	3	coral reef	45	? (A.s.)	[49]
		H2	Bali, Indonesia (A.s.)	3	A.s.	46	? (A.s.)	[49]
	III	H7	‘Indo-Pacific’ (A.s.)	1	A.s.	48	2002	[51]
	V	H4	Bali, Indonesia (A.s.)	3	coral reef	46	03/2010	this review
	VII	H12	Indonesia (A.s.)	2	coral reef	45	? (A.s.)	[49]
W Pacific Ocean	I	H1^d^	Russian coast of the Sea of Japan	1	?	52	?	[68]
		H2	Chatan, Okinawa, Japan	2	boat dock	56	03/2007	[49]
		H2	Hong Kong, China	1	flow-through seawater system	51	09/2007	this review
	III	H8	Guam, US	1	?	59	2002	[51]
	V	H4	Hong Kong, China	2	mangroves	51	03/2007	[49]
		H4	Kota Kinabalu, Sabah, Malaysia	3	boat dock	49	09/2005	[49]
		H9^e^	Lizard Island, Australia	17	coral reef (A.s.)	63	2005	[50]
		H13	Hong Kong, China	8	flow-through seawater system	51	04/2006, 09/2007	[49]
		H14	Hong Kong, China	1	flow-through seawater system	51	04/2006	[49]
		H15^c^	Shirahama, Honshu, Japan	1	rocky shore	55	09/2007	[64]
		H15^c^	Boracay, Philippines	4	stony beach	58	09/2007	[49]

Haplotypes (H1–H19) and clades (I–VII) are listed according to their oceanographic regions.

a : this haplotype was entered in Genbank as ‘*Trichoplax* sp. H12’. According to [49], [62] this sample was here renamed to ‘Placozoa sp. H17’;

b : the haplotype of the isolate ‘JM614’ is unknown, but it was described as a clade V representative in [63];

c : samples derived from ‘rock sampling’;

d : the Russian clone is highly similar to H1 but several ambiguities in the sequence, possible due to sequencing errors, prevent a clear classification;

e : originally named ‘LIZ’ in [50] but as identical to H9 sequences it was renamed in [49]. A.s. = Aquarium/holding tank sample; W = Western; E = Eastern; C = Central.

### Genetic diversity – Mitogenomics

The first complete placozoan mitochondrial (mt) genome was sequenced from *Trichoplax adhaerens* (H1, clade I; [42], [51]). It displays a combination of genomic features that are unique within the Metazoa (e.g., reduced metazoan core gene set), but also shows features linking it to single celled eukaryotes (e.g., split genes, introns and large intergenic spacers). The combination of these characteristics underlines the key position of the Placozoa in the metazoan Tree of Life. With a size of over 43 kb it is more than twice as large as the typical bilaterian mt genome, and by far the largest circular metazoan mt genome known. The characterization of mt genomes from four additional placozoans, Placozoa sp. H3 (‘BZ2423’ strain; clade II, 36 kb), sp. H4 (‘BZ49’ strain; clade V, 37 kb), sp. H8 (‘BZ10101’ strain; clade III, 32 kb) [63] and sp. H15 (‘Shirahama’ strain; clade V, 36 kb) [64]), shows that the extended size of the mitochondrial genome is a shared feature among placozoans.

The observed gene arrangements in the known placozoan mt genomes, as well as sequence analyses [63], [65], show a clear subdivision in group A (Placozoa spp. H4, H8, and H15) and group B (*Trichoplax adhaerens* and Placozoa sp. H3) mt genomes. Members of both groups are also clearly distinguished by a large inversion of a ∼20 kb fragment between *nad1* and *cox2*. Even within the two groups, translocations and/or inversion events occur suggesting that sequencing of additional mitochondrial genomes will improve the resolution of genealogical relationships, even between closely related placozoan species.

### Global distribution of genetic lineages

Placozoans have been found in all Oceans sampled to date, including the Atlantic, the Pacific and the Indian Oceans (Figure 5). The current picture shows a latitudinal distribution between 48°N to 35°S (i.e., Roscoff, Northern France and Adelaide, Southern Australia, respectively). In total, placozoans have been reported from 72 locations worldwide [2], [13], [17], [20], [21], [34], [49]–[58], [62]–[64], [66]–[79]. The most intensely studied areas are the Caribbean and the Mediterranean Sea. Other areas of intensive sampling are the central Indo-Pacific and the Western Pacific Ocean. There is very limited, or no knowledge, on the distribution of placozoans from the coasts of South America, Africa and the Indian Ocean. Placozoans have been found in the Indian Ocean at only five different sites and information from the coasts of the central Indian Ocean is completely lacking. From the West coast of Africa we have information from the Canary Islands and from Madeira, but not a single mainland coast has been sampled. In South America, placozoans have been found only in Venezuela and Brazil so far.

In summary, it can be concluded, from samplings of the last two decades, that placozoans can be found on coasts with:

- latitudes between 48°N to 35° S
- water temperatures between 10–32°C
- hard substrates
- salinity between 20–50 ppt
- low to medium wave activity in the intertidal zone

This broad range of parameters obviously indicates a wide spectrum of ecological niches that can be inhabited by placozoans.

Mapping genetic data onto the geographic distribution patterns of the placozoans reveals a striking distribution of the various genetic lineages (Figure 5). Clades I and V show large distribution ranges as both have been found in all Oceans sampled to date. Obviously clades I and V (and especially H2 and H4 within these) are able to tolerate a wide range of environmental conditions and are thus widespread (i.e., are euryoecious species). In contrast to these cosmopolitan clades, others either show a comparatively reduced latitudinal range (e.g., clade III) or are even restricted to a certain region (e.g., clade II). So far, clade II has only been found in the Caribbean and might be endemic to that region. Clade III haplotypes have so far only been found in a comparatively narrow latitudinal window from 24° North (Bahamas) to 3° South (Mombasa, Kenya). Not a single clade III specimen has been reported from the temperate Mediterranean Sea, indicating its linkage to regions with low seasonal changes. Clade IV, originally found in the Mediterranean, has recently been collected in Adelaide in the southeastern Indian Ocean, both of which are temperate areas with relatively low mean sea surface temperatures. Clade IV seems, therefore, restricted to cooler locations and probably adapted to lower temperatures than other clades.

### Taxonomic criteria

On a somewhat superficial level, the taxonomic problem we are faced with for the Placozoa might resemble a similar problem to that faced in Bacterial taxonomy [80]. In that system, only about 7000 species have been described while current estimates indicate that millions exist. Given that the current taxonomic status of the phylum Placozoa accommodates only a single species, and the information presented above indicates that there is considerable genetic, anatomical and geographic diversity, we can conclude that extensive revision of the group is needed. This situation affords us a nearly unprecedented opportunity to not only revise the group, but also make some interesting observations on the nature of taxonomy, as Placozoa become an important “taxonomic *tabula rasa*".

At this point in time we suggest that the Placozoa should fall under the aegis of the International Code of Zoological Nomenclature (see http://iczn.org/code) as Placozoa are considered animals. This set of rules is under the oversight of The International Commission on Zoological Nomenclature (ICZN). We look at the science of taxonomy as a highly hypothetico-deductive process. In this context a hypothesis of species existence is developed and all evidence related to the rejection of the hypothesis are gathered and directed towards the test of the hypothesis. Intertwined with this process is the need for a species concept, i.e. a definition of what a species is. Since sexual reproduction is probably occurring in the Placozoa [6], [9], [16], [81], [82], one might suggest that a biological species concept (BSC) could be applied to this phylum. However, determining if mating occurs between taxa, as well as determining the viability and fertility of subsequent offspring in nature are, needless to say, extremely difficult. Proxies exist for recognizing the product of this species concept, and one that we can embrace here for the Placozoa would be a diagnostic approach. Such an approach has been outlined by DeSalle *et al.*, 2005 [60] in the context of DNA barcoding and involves the use of an integrative taxonomy framework [83], [84]. In this approach, a hypothesis of species existence is erected using some starting information such as geographic isolation, or morphological differentiation. The hypothesis is tested with various forms of data, whether it be DNA sequences, anatomy, behaviour or ecology; and therefore the hypothesis is either rejected (in which case only a single species exists) or not (in which case a new species can be erected; [60]). This approach appears to be well suited to the Placozoa as ecological and habitat differences of different accessions of Placozoa differ broadly and range from euryoecious to stenoecious amongst different clades of Placozoa (as described above). In addition, the Placozoa are also geographically widespread and show different latitudinal range for the various clades, and there is also enough genetic and morphological variability to erect and test other hypotheses using these criteria.

The problem of naming higher taxa is a more difficult taxonomic task. If classical Linnean approaches are applied this problem can be resolved using, for example, sponges or even fungi as a yardstick. On the other hand, a formal code of nomenclature that does not use the Linnean system, called the International Code of Phylogenetic Nomenclature, or PhyloCode for short, can also be applied [83], [85].

## Concluding Remarks

Resolving the biodiversity of placozoans is clearly an important task. The potential evolutionary impact of this enigmatic phylum is substantial and the number of cryptic species is probably high. Half a dozen whole genome and transcriptome sequencing projects are underway, but the biology and ecological importance of placozoans are still poorly known. Worldwide sampling efforts have to be substantially increased in order to unravel the global placozoan diversity. Placozoans also represent a challenge with respect to how modern systematics can be accomplished. The large genetic diversity of placozoans is not reflected by clear morphological diversity. This observation makes applying classical, anatomical, approaches to the taxonomy of the phylum difficult. However, a combination of ecological patterns with searches for molecular characters might be an optimal approach for applying a diagnostic system to describing new placozoan species.

## Materials and Methods

Placozoans were sampled as described above. Samples were not collected at privately owned or protected areas. No specific permissions were required because of the low-level environmental impact of our collection methods, the microscopic size of placozoans, and the fact that placozoans are not CITES listed or present on any other restrictive listings, and have no commercial value. DNA isolation and fragment amplification of the mitochondrial large ribosomal subunit (16S) was performed as described [49]. Sequences of new isolates were deposited in Genbank at NCBI (http://www.ncbi.nlm.nih.gov) with accession numbers JQ924031-JQ924045. The Placozoa spp. H17, H18 and H19 sequences were aligned to the existing 16S alignment [49] using the MAFFT [86], [87] web application (at http://mafft.cbrc.jp/alignment/server/). The MAFFT-E-insi algorithm was used at default settings and the existing 16S alignment was used as structural guidance. After alignment, all ambiguously aligned positions were removed manually according to the guide alignment. The final matrix consisted of 19 sequences (for H1 to H19) with a total length of 533 nucleotide positions including gaps (see Dataset S1). Subsequent Maximum Parsimony, Maximum Likelihood and Bayesian phylogenetic inferences were performed as described [49]. To estimate the haplotype completeness a Coleman rarefaction curve [88], [89] was calculated in EstimateS v8.2 (http://viceroy.eeb.uconn.edu/EstimateS).

## Supporting Information

Dataset S1 **16S alignment used in phylogenetic analyses in** **Figure 4** **.**
(NEX)
